# Response to: Premature deaths attributed to ambient air pollutants: let us interpret the Robins–Greenland theorem correctly

**DOI:** 10.1007/s00038-017-0956-7

**Published:** 2017-03-15

**Authors:** Marie-Eve Héroux, H. Ross Anderson, Richard Atkinson, Bert Brunekreef, Aaron Cohen, Francesco Forastiere, Fintan Hurley, Klea Katsouyanni, Daniel Krewski, Michal Krzyzanowski, Nino Künzli, Inga Mills, Xavier Querol, Bart Ostro, Heather Walton

**Affiliations:** 1 WHO European Centre for Environment and Health, WHO Regional Office for Europe, Bonn, Germany; 2 MRC-PHE Centre for Environment and Health, Population Health Research Institute, St George’s, University of London, London, UK; 3 Institute for Risk Assessment Sciences, Universiteit Utrecht, Utrecht, The Netherlands; 4 Julius Center for Primary Care and Health Sciences, University Medical Center Utrecht, Utrecht, The Netherlands; 5 Health Effects Institute, Boston, USA; 6 Department of Epidemiology, Lazio Regional Health Service, Rome, Italy; 7 Institute of Occupational Medicine, Riccarton, Edinburgh, UK; 8 Department of Hygiene, Epidemiology and Medical Statistics, University of Athens Medical School, Athens, Greece; 9 Department of Primary Care and Public Health Sciences and Environmental Research Group, King’s College London, London, UK; 10 McLaughlin Centre for Population Health Risk Assessment, University of Ottawa, Ottawa, ON Canada; 11 Environmental Research Group, King’s College London, London, UK; 12 Swiss Tropical and Public Health Institute, Basel, Switzerland; 13 University of Basel, Basel, Switzerland; 14 Public Health England, Centre for Radiation, Chemical and Environmental Hazards, Chilton, Didcot, Oxfordshire UK; 15 Institute of Environmental Assessment and Water Research (IDÆA), Consejo Superior de Investigaciones Científicas (CSIC), Barcelona, Spain; 16 Office of Environmental Health Hazard Assessment (OEHHA), Research Triangle Park, USA; 17 MRC-PHE Centre for Environment and Health, and NIHR Biomedical Research Centre at Guy’s and St Thomas’ NHS Foundation Trust and King’s College London, London, UK

We thank Morfeld and Erren for their continued interest in the WHO Health risks of air pollution in Europe (HRAPIE) report (WHO Regional Office for Europe 2013). The key point of contention seems to be the interpretation of the numbers of ‘premature deaths’ associated with air pollution (or any other) exposure. In the IJPH article that is at the basis of the two letters written by Morfeld and Erren (Heroux et al. 2015), the limitations of calculating and using numbers of ‘premature deaths’ were perhaps not sufficiently explained. We elaborated on this in our first response (Heroux et al. 2016), arguing that the criticized calculation of ‘premature deaths’ produces a reasonable albeit ambiguous estimate, for which reason calculation of years of life lost is a more preferable approach. We would like to point out that the HRAPIE report really is about identification of concentration–response functions to be further used in health impact assessments, and therefore did not pretend to provide a discussion of estimating etiologic fractions. Morfeld and Erren single out the one numerical example of an impact assessment given in our paper, and that example was not a result from the HRAPIE work itself but a quote from a report from the European Commission (2013). We never intended to give the impression that these numbers refer to individually identifiable, attributable deaths, however.

The HRAPIE report itself clearly spells out the limitations of calculating ‘premature deaths’, on pages 15 and 16 (WHO Regional Office for Europe 2013). Indeed, there is a long history in the field of quantifying the mortality burden of air pollution that shows clear awareness of the complexities raised by Morfeld and Erren. For instance, the United Kingdom Committee on the Medical Effects of Air Pollutants (COMEAP) report of 2010, to which the HRAPIE report refers, contains a careful discussion on pages 5 and 70–73 of expressing mortality effects of air pollution in terms of years of life lost, and numbers of attributable or premature deaths (COMEAP 2010). Several of us were in COMEAP when it produced this report, which spells out that the mortality burden of air pollution is likely shared by many more than just the numbers of attributed deaths that are being calculated by the usual simple (RR-1)/RR formula also quoted by Morfeld and Erren. Some of us developed a similar line of arguments already back in 2007 (Brunekreef et al. 2007). As pointed out by Morfeld and Erren, Robins and Greenland (1989) showed that the (RR-1)/RR formula can underestimate as well as overestimate the attributable cases, which we have argued before as well, and therefore do not dispute.

Appreciating the limitations of death counts as a metric for quantifying disease burden due to exposure to air pollution and other risk factors, the widely cited global burden of disease exercises have since their inception quantified such burdens both in terms of the number of deaths in a given year attributable to past exposure and in terms of lost years of healthy life, or DALYs (Murray and Lopez 1999; Murray et al. 2004; Lim et al. 2012; GBD 2013 Risk Factor Collaborators 2015; WHO 2016a, b). The work of Greenland and Robins, cited by Morfeld and Erren, informed this approach.

We appreciate the opportunity for clarification of the HRAPIE report, article, and first response and agree with Morfeld and Erren that estimated burdens of disease attributed to air pollution need to be correctly interpreted in the context as explained in more detail above and in the quoted references.
